# Exploring the impact of mindfulness-based training on the well-being of physical therapists

**DOI:** 10.1017/cts.2023.666

**Published:** 2023-11-16

**Authors:** Akash Patel, Ruchi Bhargava, Gretchen Roman

**Affiliations:** 1 Physical Therapy Education Program, Midwestern University, Glendale, AZ, USA; 2 Clinical Psychology Program, Midwestern University, Glendale, AZ, USA; 3 Department of Family Medicine, University of Rochester, Rochester, NY, USA

**Keywords:** Burnout, mindfulness, physical therapy, reflective writing, well-being

## Abstract

**Introduction:**

Experimental evidence is needed to evaluate interventions that curtail burnout for physical therapists. The goal of this research was to assess the impact of mindfulness-based training (MBT) on the well-being of physical therapists. We hypothesized physical therapists would demonstrate greater work engagement, empathy, and job satisfaction, and lower depression, anxiety, stress, and moral distress following MBT.

**Methods:**

Thirteen physical therapists (10 female/3 male; 35.38 ± 9.32 years old) completed this two-arm embedded mixed-methods pilot study. The control group (*n* = 4) was followed while the intervention group (*n* = 9) completed six MBT sessions over 3 months. Sessions were assigned a representative topic area (meaning in physical therapy, situational- and self-awareness, compassion fatigue/burnout, implicit biases, establishing boundaries and managing conflict, self-care) with relevant reflective writing, small group discussions, and mindfulness strategies. Non-parametric statistics compared quantitative outcomes across and within groups, and a thematic framework matrix was established by way of qualitative description for data analysis.

**Results:**

Physical therapists in the intervention group had improved pre- to post-scores for work engagement, mental health, and moral distress (*p* ≤ 0.043). *Inability to effect change* contributed to compassion fatigue/burnout, whereas *difficulty prioritizing self/limited personal time* impeded self-care. “*I realized how easy it is to get caught up in life and in helping out those around you, you completely forget to take time to check in with how you are doing (Physical Therapist 3)*.”

**Conclusion:**

Implementing an MBT program demonstrates promise and may benefit the well-being of physical therapists while simultaneously enhancing employee retention and improving patient care.

## Introduction

Burnout is a collection of symptoms, including exhaustion from one’s profession, loss of motivation, and decreased sense of accomplishment within the workplace [1]. Healthcare workers have reported a high prevalence of burnout compared to those in other industries [2,3]. The number of studies investigating burnout in healthcare has increased over the past few decades; however, most have focused on nurses and physicians [4–8]. Of those studies, some have explored interventions for mitigating burnout [6–8]. There are substantially fewer studies examining burnout and interventions to address well-being experienced by other healthcare professionals, including physical therapists.

Many physical therapists resort to leaving the profession due to stress and burnout [9,10]. In a 2022 report on the State of Rehab Therapy, rehabilitation therapy employees demonstrated greater turnover when compared to healthcare in general with physical therapists and physical therapist assistants reporting the most turnover [11,12]. Seventy percent of rehabilitation therapy professionals had thoughts of a professional change with 27% either considering a non-clinical role or leaving the field entirely [11]. Physical therapists become morally distressed from moral injury (unable to provide high-quality care and healing), which ultimately leads to burnout symptoms involving depression, exhaustion, or anxiety [13]. High patient load, long work hours, and low salary were the top three contributors to 35% of therapy professionals feeling burnout [11]. In some institutions, physical therapists feel compelled to act unethically, including overbilling and high productivity requirements leading them to burnout [14]. Unfortunately, with increases in physical therapists’ responsibilities, complexities of the healthcare system, institutional constraints, and demand for physical therapy, symptoms of burnout and moral distress will likely proportionately increase.

As of 2020, the average balance for student loan debt of recent physical therapy graduates was more than $116,183 with the average salary ranging from $50,000 to $99,000 depending on location [15]. Student loan payback options were the most infrequently reported strategy used for promoting employee retention [11]. The growing loan debt-to-salary ratio along with the moral distress experienced in the clinic further compounds burnout symptoms in physical therapists and poses a concern for overall well-being [13,14,16]. As a result, the quality of care given to patients may be suffering, potentially leading to higher costs for the healthcare system. Despite all this, there is a lack of research addressing these issues within the physical therapy profession. The few studies that exist add to the available literature and confirm the experience of burnout among physical therapists; however, these studies employ cross-sectional surveys and do not provide interventions to help mitigate burnout [9,10]. Further studies exploring specific interventions to ward off burnout and promote well-being in physical therapists are warranted.

The goal of this pilot study was to assess the impact of mindfulness-based training (MBT) on the well-being of physical therapists. We sought to introspectively explore personal and clinical experiences, as well as evaluate well-being outcomes. We hypothesized that participants in the intervention group receiving MBT would demonstrate higher work engagement, empathy, and job satisfaction, and lower depression, stress, anxiety, and moral distress compared to participants in the control group.

## Materials and methods

This two-arm embedded QUAN(qual) mixed-methods pilot study (AZ 1258) was approved by the Institutional Review Board at Midwestern University in Glendale, AZ. The secondary analysis of the deidentified qualitative data (STUDY00008177) was exempt from review by the Research Subjects Review Board at the University of Rochester in Rochester, NY.

### Participants

Community physical therapists were recruited through social media, established networks of the study team, and flyers permitted to be displayed in physical therapy clinical sites. Licensed physical therapists working full-time in a clinical setting and treating patients for at least 1 year were included.

### Design and procedures

**Figure 1.**
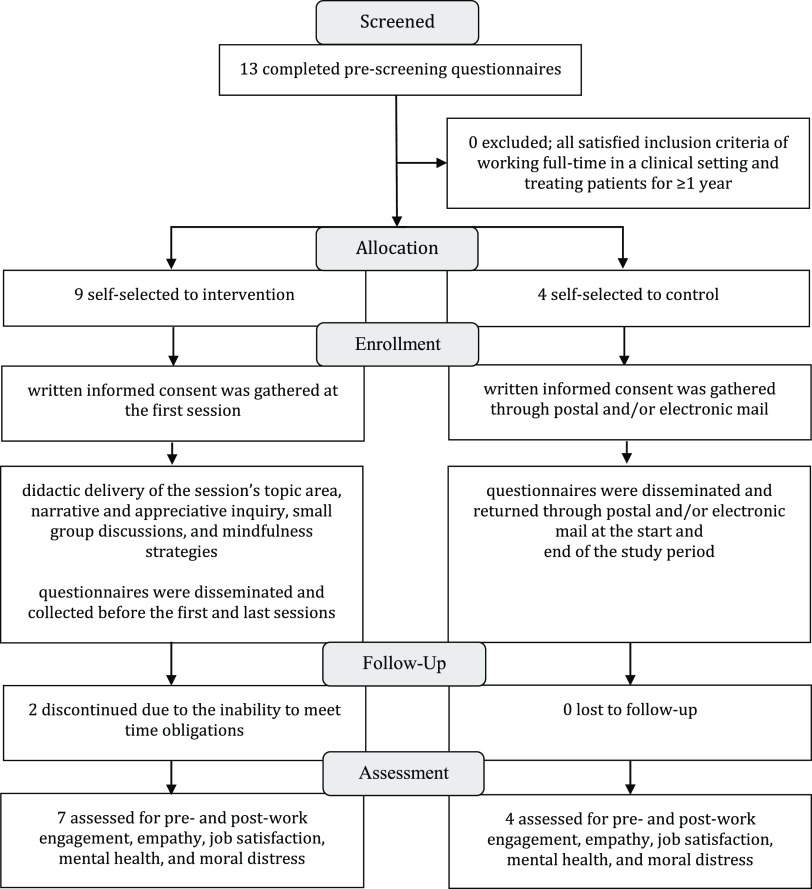
Flow diagram of the methods [49].

After expressing interest in the study, prospective participants completed a pre-screening questionnaire to determine satisfaction of the inclusion criteria (Fig. 1). All participants self-selected to take part in either the intervention or control group based on availability and voluntarily provided written informed consent. Participants who were able to satisfy the regular, ongoing commitment to the MBT curriculum opted to participate in the intervention group and those who still wanted to participate in the study, however, were unable to meet the time obligations of the intervention opted for the control group. The Utrecht Work Engagement Scale [17,18], Toronto Empathy Questionnaire (TEQ) [19], Minnesota Satisfaction Questionnaire [20,21], Depression, Anxiety, Stress Scale (DASS-21) [22–25], and Measure of Moral Distress for Healthcare Professionals (MMD-HP) [26,27] were disseminated and returned through postal and/or electronic mail for the identified control participants at the start and end of the study period, and before the first and last MBT sessions for the intervention participants (Table 1).

**Table 1.** Description of tools used to measure the well-being outcomes of physical therapists

Outcome	Tool	Description
Work engagement	Utrecht Work Engagement Scale (UWES-17)	The UWES-17 was used to quantify participants’ engagement in their work as physical therapists performing administrative duties and patient care. This tool separately measures the work engagement dimensions of vigor (six questions), dedication (five questions), and absorption (six questions) using a Likert scale ranging from zero to six (zero=never; six=always) [17,18]. The participant-rated responses are totaled within each dimension and divided by the total number of questions pertaining to that dimension. Higher scores reflect more occupational involvement while displaying higher energy levels to meet higher occupational demands. Lower scores demonstrate the contrary. Significant construct validity and acceptable to good internal consistency have been measured [18]. Permission to use the UWES-17 was granted by Wilmar B. Schaufeli, PhD, a professor of work and organizational psychology at Utrecht University and co-developer of the UWES scale
Empathy	Toronto Empathy Questionnaire (TEQ)	The TEQ scale assesses feelings of emotional investment displayed during interactions at work. This tool includes 16 questions and is a brief, reliable, and valid instrument [19]. The sum of the scores, eight positively worded (zero=never; four=always) and eight negatively worded (four=never; zero=always) questions, is used to determine the participant’s level of empathy. With the maximum score being 64, higher scores indicate a higher level of empathy and vice versa. Permission to use the TEQ was granted by Nathan R. Spreng, PhD, an Associate Professor in the Department of Neurology and Neurosurgery at McGill University and developer of the TEQ [19]
Job satisfaction	Minnesota Satisfaction Questionnaire (MSQ-20)	Each participant’s level of job satisfaction was measured using the MSQ-20. This tool measures a worker’s occupational satisfaction and was obtained without charge or permission courtesy of Vocational Psychology Research in the Department of Psychology at the University of Minnesota [20]. Of the 20 questions on the questionnaire, 6 measure extrinsic, 12 measure intrinsic, and 2 measure overall job satisfaction garnering three measures in total from the MSQ-20. Questions were scored based on a five-point Likert scale (one=very dissatisfied; five=very satisfied). The maximum scores for each level of job satisfaction are as follows: 60 for intrinsic, 30 for extrinsic, and 100 for general job satisfaction. Higher scores indicate higher satisfaction and vice versa. Valid and reliable psychometric properties were determined by Martins and Proenca [21]
Mental health	Depression, Anxiety, Stress Scale (DASS-21)	Depression, anxiety, and stress were measured using the DASS-21 scale. This tool has separate depression, anxiety, and stress subscales, each measured by seven questions for a total of 21 questions. Responses from zero to three (zero=never; three=almost always) are summed and multiplied by two with higher scores indicating worse depression, anxiety, or stress. The overall normal score on the DASS-21 ranges from zero to 30. Subscale scores of 0–9, 0–7, and 0–14 indicate levels normal of depression, anxiety, and stress, respectively [22,23]. Subscale scores greater than the normal range can be categorized from mild to extremely severe [23]. Acceptable to good internal consistency was measured for each subscale and for the overall scale [24]. Permission was not required for the use of the DASS-21 as it is publicly available for academic and research purposes from the School of Psychology at the University of New South Wales [25]
Moral distress	Measure of Moral Distress for Healthcare Professionals (MMD-HP)	Moral distress has important implications regarding the retention rates of healthcare workers and their satisfaction with their careers and the quality of patient care [26]. Moral distress was measured using the MMD-HP, which contains 27 items. Dimensions of frequency and level of distress were addressed for each item from zero to four (zero=never; four=very frequently). The score for each item is obtained by calculating the product of the two dimensions for a total potential score of 16 per item with the total being the sum of 27 items. Higher scores indicate higher moral distress and vice versa. Excellent reliability and validity have been analyzed [27]. Permission was not required for use of the MMD-HP as noted by Dr Ann Hamric, PhD, RN, FAAN, a former Professor and Associate Dean of Academic Programs at Virginia Commonwealth University and co-creator of the MMD-HP scale

The intervention group met for a total of six sessions. Participants were allowed one absence due to unexpected life events; however, they were asked to complete the missed study activities remotely. For any participant who was unable to meet the study’s time obligations, any specific measures that were willingly completed and data collected up to the point of the participant withdrawal were still included in the analysis. Roughly 2 weeks between sessions were allotted, allowing for practice of the introduced mindfulness strategies. Performance of the mindfulness strategies was not recommended at a specific frequency and how often the participant employed the strategies was not recorded. Study investigators discussed the most pertinent topic areas and adapted an educational program in mindful communication [6] to the unique needs of community physical therapists. Once a schedule of predetermined dates was established, each session was assigned a representative topic area (Table 2). Two of the intervention topics (being with suffering or end-of-life care and attraction in the clinical encounter) in the previous work [6] were deemed to be not as applicable; thus, study investigators abbreviated the intervention by reducing the number of study sessions from eight to six. The duration of each session was also changed from 150 to 90 minutes to allow for minimal disruption over the 3-month study period. The topics and narrative prompts that were more specific to medicine in the previous work [6] were adapted to the context of physical therapy. For example, the topic of “awareness of pleasant and unpleasant sensations, feelings, or thoughts” was re-worded to “situational- and self-awareness” and the associated narrative prompt “a pleasant or an unpleasant experience during clinical work and its effect on the patient-physician relation” was changed to “…pleasant or unpleasant thoughts or feelings you may have experienced during clinical practice. Did these sensations have any effect on your professional relationship with the patient?” The topic of “meaning in medicine” was also changed to “meaning in physical therapy.” Mindfulness was woven throughout each session, which consisted of didactic delivery of the topic area, narrative and appreciative inquiry, small group discussions, and specific mindfulness strategies. The control group received no intervention and was followed over the roughly 3-month study period.

**Table 2.** Adapted mindfulness-based training (MBT) curriculum [6] for physical therapists

Session	Topic area	Mindfulness strategy	Narrative prompt
			Write a brief story about
1	Meaning in physical therapy/introduction	Mindfulness walking	A meaningful clinical encounter. What made it meaningful? What did you learn from the encounter? What personal characteristics did you contribute?
2	Situational- and self-awareness	Five senses and body scan	Pleasant or unpleasant thoughts or feelings you may have experienced during clinical practice. Did these sensations have any effect on your professional relationship with the patient?
3	Compassion fatigue/burnout	Review of mindfulness strategies	A time when you were emotionally exhausted from clinical work and felt a low sense of accomplishment
4	Implicit biases	Deep breathing and visualization	A clinical experience that was completely different than what you expected
5	Establishing boundaries and managing conflict	Review of mindfulness strategies	A time when you were able to cultivate a healing relationship while maintaining professional boundaries
6	Self-care/summary	Guided imagery	A time when you had to prioritize self-care over the care of others or the care of others over self-care your reflection on the last 3 months

### Intervention

#### Didactic delivery of the topic area

The representative topic area for each session was introduced didactically (Table 2) for the first 10 minutes of each session by one of the study investigators. Introduction of the study was performed at the beginning of session one and a summary of the prior sessions was given at session six. Any participants who missed a session were sent information about the specific session’s didactic topic area via electronic communication.

#### Narrative and appreciative inquiry

Participants were issued designated composition notebooks, which served as a journal for the duration of the study. Study journals were secured in a study investigator’s office between sessions. During the second 10 minutes of each session, participants were encouraged to introspectively explore how the session’s narrative prompt (Table 2) resonated with their personal and clinical experiences through reflective writing. Participants were then guided to apply the appreciative inquiry framework to the session’s topic area. The appreciative inquiry framework consisted of the following prompts: discovery, dream, design, and destiny, or the 4Ds. These prompts encouraged participants to, respectively, consider “what is,” “what might be,” “what should be,” and “what will be [28].” A total of 20 minutes of appreciative inquiry (5 minutes per each framework prompt) was performed in each session. Any participants who missed a session were asked to complete the session’s narrative and appreciative inquiry reflections via electronic communication, which was later printed and added to the participant’s study journal for reference.

#### Small group discussions

After completing the narrative and appreciative inquiry, the intervention group was divided into three small groups of two to three participants each facilitated by one of the study investigators for 30 minutes. Study investigators made every effort to establish a secure environment, where the participants would feel safe sharing their work experiences. During the MBT curriculum development, the study team agreed on different tactics to prompt natural discussion in small groups. Mainly, participants were encouraged to express their thoughts, feelings, and emotions based on the session’s topic area and, if comfortable, share what they wrote in their journals during the narrative and appreciative inquiry reflections. Out of respect for one another’s privacy, participants were asked to keep the information shared and discussed throughout the MBT curriculum contained within the study setting.

#### Mindfulness strategies

Following the small group discussions, specific mindfulness strategies concluded each session and were facilitated by the clinical psychologist investigator for 20 minutes. Such strategies were designed to help participants create simple anchors when needing to bring attention back to the present moment or become aware of the afferent signals from within the body to better respond to stressful internal stimuli. Mindfulness walking [29,30] was introduced during the first session (Table 2). Participants were instructed to walk around our local campus community while simultaneously exploring their senses, such as observing the color of the buildings, the smell of the air, or any sounds outside. The five senses exercise [31,32] and the body scan [33,34] were introduced during the second session. The five senses exercise asked participants to use sight to observe their surroundings, notice specific tastes in their mouths, detect scent and sound in the room, and identify the touch of their hands, feet, and other body parts upon the surface in which they were sitting. During the body scan, participants were invited to close their eyes and verbally guided to observe physical sensations from their feet to their heads. Mindful walking, five senses, and body scan were reviewed in the third session. Deep breathing [35,36] and visualization [37,38] were introduced during the fourth session. Participants were instructed to inhale for 4 seconds, hold their breath for another 4 seconds, and then exhale for 6 seconds for a minimum of 2 minutes. Participants were also provided with handouts of visualization techniques, like a serene beach scene, blue light, a ball of yarn, liquid quiet, and a double-paned window. Mindfulness walking, five senses, body scan, deep breathing, and visualization were reviewed in the fifth session. Guided imagery [39] was introduced during the sixth and final session. The clinical psychologist investigator read a narrative script that guided participants through scenic imaginations. In addition to engaging in the mindfulness strategies as a component of each study session, participants were advised to trial the different strategies while at work in between study sessions. Any participants who missed a session were sent information about the specific session’s mindfulness strategies via electronic communication.

### Data analysis

Pre- and post-questionnaires were collected from both groups approximately 3 months apart. The questionnaires were dispensed and collected at the same time for the intervention group and at different times for the control group as participant recruitment was ongoing. All questionnaires were appropriately scored and scores were compiled (Microsoft Excel, v.2021, Microsoft, Redmond, WA). Descriptive statistics (mean±SD) were calculated from the raw data. Because normality assumption was not achieved, non-parametric statistics were used to compare across and within groups. Separate Mann–Whitney U tests were used to evaluate pre-questionnaire scores across groups, and separate Wilcoxon signed-rank tests were used to compare pre- to post-questionnaire differences within each group. Chi-square tests evaluated differences across groups in the covariates of age, gender, years of clinical experience, and total hours worked per week. With significance as *p* < 0.05, all quantitative statistical analyses were performed using SPSS (v.29, IBM, Armonk, NY).

A thematic framework matrix was established by way of qualitative description using MaxQDA 2020 (v20.4.2, VERBI, Berlin, Germany) for the qualitative analysis [40,41]. Because of the mixed-methods embedded QUAN(qual) research design for this study, we opted for the straightforward description of the phenomena using this approach [42]. One study investigator (GR) manually transcribed the reflective writing from the journals into electronic format. Two investigators (AP and GR) separately reviewed the transcriptions and initially coded the qualitative data within each topic area. Over a period of weeks, AP and GR worked together to triangulate common themes and subthemes relating to the narrative and appreciative inquiry reflections until reaching a consensus. After three total hours of reflective writing across the six MBT sessions for each participant, saturation occurred when no new themes or subthemes emerged in their responses to the presented narrative prompt or across the 4Ds for each session’s topic area. Finally, quantitative and qualitative data were integrated to accentuate any concordant or discordant findings [43].

## Results

### Participants

Thirteen physical therapists (10 female/3 male; 35.38 ± 9.32 years old) working across nine different healthcare organizations completed this pilot study. Nine participants (8 female/1 male; 37.22 ± 10.79 years old) were involved in the intervention group. Intervention participants had 9.50 ± 9.90 years of clinical experience and worked 41.67 ± 8.72 total hours per week. Four intervention participants worked in outpatient settings, two in inpatient settings, one in skilled nursing, and two across multiple settings. Four participants (2 female/2 male; 31.25 ± 2.06 years old) were involved in the control group. Control participants had 5.25 ± 2.75 years of clinical experience and worked 40.00 ± 0.00 total hours per week. One control participant worked in an outpatient setting, two in inpatient settings, and one in skilled nursing. There were no differences across groups in age (*p* = 0.195), gender (*p* = 0.079), years of clinical experience (*p* = 0.234), and total hours worked per week (*p* = 0.221). Clinical specialty by the American Board of Physical Therapy Specialties was maintained by four participants (two Geriatric Clinical Specialists; two Orthopaedic Clinical Specialists) in the intervention group and by one participant (Orthopaedic Clinical Specialist) in the control group. There was a 100% retention rate in the control group, whereas two of the participants in the intervention group were unable to complete the study due to the time obligations yielding a retention rate of 84.6% (Fig. 1). Each participant in the intervention group missed one of the six sessions.

Pre-scores across intervention and control groups were compared for the well-being outcomes. For work engagement, no differences were analyzed for pre-vigor, dedication, and absorption (*p* ≥ 0.437). For empathy, no differences were analyzed across groups for pre-TEQ (*p* = 0.815). No differences were analyzed across intervention and control groups for pre-overall, intrinsic, and extrinsic (*p* ≥ 0.392) job satisfaction. For mental health, no differences were analyzed for pre-overall, depression, and anxiety (*p* ≥ 0.120). There were differences detected across groups for pre-stress (*p* = 0.042) with the intervention group reporting greater pre-stress (14.44 ± 8.99) than the control (7.50 ± 1.91) (Table 3). Finally, no differences were measured across groups for pre-moral distress (*p* = 1.000).

**Table 3.** A comparison of pre- to post-scores within intervention and control groups (mean±SD)

Outcome		Intervention group		p-value	Control group		p-value
		Pre	Post		Pre	Post	
UWES-17	Vigor	4.17 ± 0.71	4.74 ± 0.59	0.063	4.37 ± 1.38	3.87 ± 1.04	0.144
	Dedication	4.40 ± 0.64	4.97 ± 0.69	0.042^*^	4.55 ± 1.19	4.30 ± 1.14	0.102
	Absorption	4.00 ± 0.70	4.83 ± 0.78	0.018^*^	4.01 ± 1.15	3.54 ± 0.98	0.109
TEQ		48.78 ± 5.09	52.86 ± 5.43	0.075	49.75 ± 5.06	48.75 ± 4.57	0.157
MSQ-20	Overall	69.43 ± 8.72	70.00 ± 10.54	1.000	69.00 ± 9.83	64.00 ± 3.74	0.273
	Intrinsic	49.43 ± 4.47	50.00 ± 4.86	0.786	48.75 ± 6.08	46.50 ± 3.42	0.285
	Extrinsic	20.00 ± 5.57	20.00 ± 6.30	0.915	20.25 ± 4.35	17.50 ± 3.11	0.141
DASS-21	Overall	24.67 ± 15.30	11.71 ± 4.39	0.042^*^	14.00 ± 5.42	11.75 ± 5.56	0.465
	Depression	6.67 ± 5.29	1.71 ± 2.43	0.043^*^	4.50 ± 2.52	2.75 ± 1.89	0.285
	Anxiety	3.56 ± 2.96	1.71 ± 2.14	0.236	2.00 ± 1.63	2.75 ± 2.22	0.593
	Stress	14.44 ± 8.99	8.29 ± 4.07	0.138	7.50 ± 1.91	6.25 ± 5.32	0.715
MMD-HP		107.78 ± 40.09	69.71 ± 55.65	0.043^*^	94.75 ± 63.39	94.75 ± 45.19	0.715

UWES-17 = Utrecht Work Engagement Scale; TEQ = Toronto Empathy Questionnaire; MSQ-20 = Minnesota Satisfaction Questionnaire; DASS-21 = Depression, Anxiety, Stress Scale; MMD-HP = Measure of Moral Distress for Healthcare Professionals.

* =*p* < 0.05.

### Quantitative outcomes

Pre- to post-scores within the intervention group and the control group were compared for the outcomes of work engagement, empathy, job satisfaction, mental health, and moral distress. Notable work engagement differences were observed within the intervention group for pre- to post-dedication (*p* = 0.042) and absorption (*p* = 0.018) (Table 3). No changes were measured within the intervention group for pre- to post-vigor, and no changes were observed within the control group for any of the pre- to post-work engagement outcomes. The intervention group had an increase in dedication and absorption of 11.47% and 17.18% while the control group had a decrease of 5.49% and 11.72%, respectively. Although significant changes were not detected within the intervention or control groups for pre- to post-empathy, the intervention group had a 7.72% increase in empathy compared with a 2.01% decrease in the control group. No changes were measured within the intervention or control groups for pre- to post-overall, intrinsic, or extrinsic job satisfaction. Notable differences were observed within the intervention group for pre- to post-overall mental health (*p* = 0.042) and depression (*p* = 0.043). No changes were measured within the intervention group for pre- to post-anxiety or stress, and no changes were observed within the control group for pre- to post-overall, depression, anxiety, or stress. The pre- to post-overall mental health improvement in the intervention group was much larger (52.53%) when compared with the control group (16.07%). In addition, the pre- to post-depression improvement in the intervention group was much larger (74.36%) when compared with the control group (38.89%). There were notable changes measured within the intervention group (*p* = 0.043), and no changes were observed within the control group from pre- to post-moral distress. The intervention group had a 35.32% reduction in moral distress compared with a 0% change in the control group.

### Qualitative outcomes

There were several important themes and subthemes that emerged in response to the narrative prompts (Table 4). *“My ability to work with this patient population, to have Parkinson’s Disease not be an end to life but something to work through, allowed me to help this patient help himself (Physical Therapist 1)”* was representative of the *regaining independence* theme for the topic area of meaning in physical therapy in session one. *Mobility improvements in patients with dementia*, *patient gratitude*, and *explaining the why behind treatment to ensure a successful discharge* were other overarching themes in session one. *Keeping an open mind*, *frustration when patients are not receiving the needed care*, and *wasted time and energy* were themes for the topic area of situational- and self-awareness in session two. For session three, the topic area of compassion fatigue/burnout elicited the theme of *existential struggle when patients are non-adherent*, “*I will have days at work where I feel like my buttons are pushed to the last point. I’ll have patients who will not participate or complain the whole time or something similar to where I feel like there’s nothing I can do to help anyone (Physical Therapist 9).” Increasing hours/workload*, *inability to effect change (lack of support from referring provider)*, and *infiltration into personal life* were also themes in response to the narrative prompt for session three. *“There are many incidents when I review a patient chart and I hypothesize the type of person that will walk through the door. Due to my biases and past experiences, however, I am sometimes extremely off (Physical Therapist 3)”* reflected the *aging patients’ level of function* theme for the topic area of implicit biases in session four. Other themes in session four were *secondary gain* and *adapting to differences*. *Amicable collaboration*, *diffusing difficult situations*, and *patients who want personal relationships* were the themes for the topic area of establishing boundaries and managing conflict in session five. Lastly, in session six, the themes in response to the narrative prompt for the topic area of self-care were *sacrifice with guilt*, *actionable self-care*, *work-life integration*, and the *effects of stress and anxiety on personal health*.

**Table 4.** Reflective writing in response to the narrative prompt

Session	Narrative prompt	Topic area	Theme	Exemplar quote
1	A meaningful clinical encounter. What made it meaningful? What did you learn from the encounter? What personal characteristics did you contribute?	Meaning in physical therapy	Mobility improvements in patients with dementia	He [the patient] was so appreciative to be out of bed and moving, even though he still couldn't comprehend why his leg hurt and wouldn't move. Dementia is so awful but working with him was so rewarding. Using everything we learned to help someone who doesn't have the capacity to help himself (Physical Therapist 5)
			Regaining independence	My ability to work with this patient population, to have Parkinson’s Disease not be an end to life but something to work through, allowed me to help this patient help himself (Physical Therapist 1)
				I feel lucky that I get to help people get back to the life they had before (or as close as possible to it) and sometimes even getting them stronger and more independent than before their hospitalization (Physical Therapist 9)
			Patient gratitude	When I first started working with the patient, I wasn't sure how much progress she would make or if she had very much potential. The more we worked together, the more she trusted me, and the more we could achieve. Ultimately, she went from bedbound to ambulatory. She cried upon discharge because she was so thankful and called me a friend. I believe it was my consistency, dedication, friendliness, and positive attitude that helped to accomplish this and make it such a meaningful experience (Physical Therapist 6)
			Explaining the why behind treatment to ensure a successful discharge	A recent clinical encounter I found to be meaningful took place with a patient who had multiple bouts of recent therapy but was not having much carryover or success resulting in him frequently returning back on services. I had not been working at the clinic prior to this, so I had not known about his past therapy failures. After our time together, he finally achieved his goals and was able to return to his prior level and stay off of therapy for greater than six months. My capacities were based on foundational knowledge, in turn teaching him how to apply these to his home program. He was able to progress because he finally understood why he had been asked to do all of the exercises in the past and then it made him able to achieve his goals (Physical Therapist 7)
2	Pleasant or unpleasant thoughts or feelings you may have experienced during clinical practice. Did these sensations have any effect on your professional relationship with the patient?	Situational- and self-awareness	Keeping an open mind	Prior to a session, I expected an unpleasant experience. I even complained to co-workers. They [co-workers] were shocked because they had never heard me complain about a patient interaction before. That made me feel good and I entered the session with an open mind. Pleasant experiences make for creative ideas and/or solutions, in turn making for a more productive treatment session (Physical Therapist 8)
			Frustration when patients are not receiving the needed care	A patient came into the clinic after having been treated at another clinic and was referred for post-operative knee surgery (total knee arthroplasty). She was dependent in all transfers and presented with signs and symptoms consistent with a cerebral vascular accident, which, per her report, began three weeks prior. She was living alone and had been treated without concern for this new onset of sudden weakness. After speaking with her son, he was not checking on her regularly and angry with his mother for being lazy. He [the son] was not willing to stay with his mother or hire assistance and she clearly was not safe to be home alone. I also spoke with the surgeon. He [the surgeon] had little interest in her sudden onset of left-sided weakness. I had no previous medical history of the patient secondary to her being out of network. It was very frustrating for me to know this patient was not receiving the care she deserved or needed (Physical Therapist 3)
			Wasted time and energy	Several years ago, I had a patient who came to our outpatient neurological/vestibular clinic for care related to a concussion. The patient complained of a wide variety of symptoms including neck pain, dizziness, imbalance, and inability to care for family and to work. The patient got himself to the appointment but then, upon standing in the waiting room, he was nearly unable to stand and walk to the treatment room. By the end of each session, he would seem to be better and we would feel like we were making progress only to have the exact same thing happen the very next session. I was very upfront with the patient about this difference between arriving fine, leaving fine but how little we were getting done each session and suggested he return to his medical doctor and also discussed with the medical doctor about getting mental health/psychology services involved as this seemed to be a bigger issue than physical. Then, the patient never returned. I felt unpleasant about putting so much effort into the patient/patient care and then, he left and never returned. The feeling was one of being unsatisfied and “unwanted,” like the patient didn't care what we were trying to do. It felt like my time and energy were wasted (Physical Therapist 7)
3	A time when you were emotionally exhausted from clinical work and felt a low sense of accomplishment	Compassion fatigue/burnout	Existential struggle when patients are non-adherent	I worked with a patient to improve his function, so he could continue living in his own home. He “cycled through” the clinic one to two times per year after that not having followed through with any of the teaching (e.g., home program, community-based activity: social and physical). It was very difficult to maintain a positive front and I sort of dreaded seeing them show up on my schedule (Physical Therapist 2)
				It gets hard to care about patients who keep coming to the hospital for the same thing and won't change or won't take advice. Physicians that ask for a consult, but obviously don't care about the recommendation. It makes me feel like, what is the point of this job? Feeling like no matter how much or fast I work, it doesn't matter to the hospital/department. It is never enough (Physical Therapist 5)
				I will have days at work where I feel like my buttons are pushed to the last point. I’ll have patients who will not participate or complain the whole time or something similar to where I feel like there’s nothing I can do to help anyone. Oftentimes, I’ll experience frustration, anger, and disappointment that carries over to my personal life. It will affect my relationship with my partner, my sleep, and my stress and anxiety levels (Physical Therapist 9)
			Increasing hours/workload	I have been experiencing burnout on some level over the last several months secondary to increasing hours over the last year. It hasn't affected my patient care, but negatively impacts my ability to document, stay focused outside of care, and my home participation. I’ve been working on maintaining awareness of my emotions, where they come from, and what they’re truly reflective of to avoid misplacing them. Also, mindfulness to address my general fatigue, emotional and physical (Physical Therapist 4)
				Over the past few months, I was becoming more and more emotionally exhausted from the intense workload of 85% productivity day in and day out. It was becoming draining for me and I was tired of working at a pace I didn't feel was sustainable and reasonable for me. I was dreading long days of patient care, something I used to love (Physical Therapist 6)
			Inability to effect change (lack of support from referring provider)	I’m currently working with a patient who is having severe pain status post approximately three weeks from a total knee arthroplasty. I’m doing all I can to increase range of motion and decrease pain while trying to avoid manipulation under anesthesia. However, she is in a lot of pain, seeing multiple specialists, and still not improving. I thought I might be missing something, like an allergic reaction to the metal implant, and sent her back to the surgeon. The doctor sent back a script stating “more aggressive physical therapy to increase knee range of motion” for ANOTHER month. It is now going on 12 weeks status-post with no change. I’m at my end, emotionally, she is 10/10 pain all of the time and I haven't made a difference at all (Physical Therapist 1)
			Infiltration into personal life	I entered into physical therapy with the hope of helping others, and when my patients don't get better, I worry I am not giving the optimal care that could improve symptoms. When I have a really tough patient or a patient who wants to see a different therapist, I will think about them at home and it begins to affect my personal life and sometimes sleep. This is also an issue I have when I feel my patient is in an unsafe environment or has medical issues that could be life-threatening. I always feel like “what did I do wrong” or “what can I do better next time.” (Physical Therapist 3)
4	A clinical experience that was completely different than what you expected	Implicit biases	Secondary gain	Having a patient come to therapy after being seen at other physical therapy clinics happens often. I recently had a patient return to me after “not getting any better” from going to her doctor’s, then her surgeon suggested physical therapy. She was very pleasant and it turns out, she had an undiagnosed cerebral vascular accident/transient ischemic attack. I asked her go to a neurologist and also, get fitted for an ankle-foot-orthosis due to her drop foot. She was so happy that someone listened to her and “helped her.” I thought this patient was “physical therapy shopping” or trying to get something (secondary gain) because I’d experienced patients in the past when I was their third or fourth physical therapist for the same issue. However, she really thought no one was addressing her foot “turning out” and that’s why they didn't “help her.” Now, I have no idea if the other physical therapists noted her drop foot because I don't have their notes, but at least the patient didn't understand that she needed an ankle-foot-orthosis until she saw me. I was able to help her after all, not “physical therapy shopping” as I initially thought (Physical Therapist 1)
				Shortly after graduating, I had a patient who seemed very motivated to get back to work. He spoke about taking care of his family and how much he loved his job. After a couple weeks, he reported little to no improvement with 10/10 pain. Objectively, he was great but continued to state he couldn't work due to his pain. I found out from another source he had a lawsuit pending and was working a second job. I was very surprised at this because I was completely bought in, however, Waddell sign was positive and there were no physiologic signs (Physical Therapist 4)
				I have perpetual biases against chronic pain patients. I have the perception sometimes that they may be resistant to physical therapy and movement. Sometimes they will be open with the fact that they are just at physical therapy to qualify for their pills. Other times, I get surprised by their willingness to try and work with me (Physical Therapist 5)
			Aging patients’ level of function	There are many incidents when I review a patient chart and I hypothesize the type of person that will walk through the door. Due to my biases and past experience, however, I am sometimes extremely off. I was reviewing the chart of an 86-year-old gentleman with low back pain. I was anticipating to see someone who was a fall risk, typical stenotic presentation, pain with extension-biased activities. The gentleman turned out to be an avid biker and continued to run several times a week. The functional goals and activities were way different than my original hypothesis. My initial prognosis was poor secondary to his age, but he ended up doing great with therapy. This situation allowed me to re-evaluate my biases and what patients/the human body is truly capable of doing (Physical Therapist 3)
			Adapting to differences	I can think of a time when a patient was saying all kinds of things that I disagreed with and found offensive. From that point on, I tried not to work with him because I had developed this negative bias towards him. One day, I had to see him for his discharge. I was really dreading it. However, he was very nice to me the entire session and we had a great conversation about something other than politics. I think it is easy to develop a negative bias towards those who express different opinions than we hold, but it is important to remember that they are still people and are worthy of 100% of our care, regardless of our differences politically (Physical Therapist 6)
				An example of a “a clinical experience that was completely different than what you expected” occurred with a repeat client at our clinic. This was my first time seeing them/treating them but the other staff members had “filled me in.” They told me the person was lazy, didn't like to participate, would come, and then quit showing up after a while only to return again in a couple months with the same problem. I went into the evaluation with an attitude and bias that the individual was going through the motions and that what I was going to do/say likely didn't matter much since the patient had already been seen and more or less failed treatment. I was biased from my colleagues’ information. Instead, however, this patient and I really got along and clicked. Unlike his previous times, he came consistently and finished his treatments and care. He improved and stayed out of the clinic for twice as long as usual after we were finished, and when he returned had actually maintained some of what we had worked on. The situation highlighted how important it is to give every patient a fair shot even if they are returning or had not done well in the past. Whether it was me or a change in him, something clicked that time around. Had I not been willing to adapt within our session and recognize his willingness, I would have written him off before we ever really started (Physical Therapist 7)
5	A time when you were able to cultivate a healing relationship while maintaining professional boundaries	Establishing boundaries and managing conflict	Amicable collaboration	A patient was being seen status-post total shoulder arthroplasty due to a fall. His script was for shoulder protocol, but his wife wanted physical therapy to do balance work. I set the goals with the patient and the patient agreed to do shoulder rehabilitation first and then, focus on balance afterward. However, his wife kept pushing the balance but wouldn't stay for the sessions. Finally, after his second progress report, she stayed and listened to why I was treating the shoulder first. Once she understood, the conflict was resolved. I treated the patient’s total shoulder arthroplasty with great results and then, we focused on his balance. I had to say “no” to treating his shoulder and balance deficits at the same time, but, ultimately, I was able to give him good care for both (Physical Therapist 1)
				A patient who had been coming to the clinic for quite some time wasn't making the progress expected. We had already extended the plan of care to allow for more time once previously. We were faced with the end of the treatment and goals had not been met. The patient wanted to continue. I had to establish boundaries, as it came to light over the last few weeks that the patient was not adhering to their home program, which was likely causing the slow progress. The patient was content to just keep coming and not do anything at home but I had to make sure the patient knew this was the last time we would extend the plan and that I was only willing to see them once a week because they needed to do the work on their own. The patient was slightly miffed and upset but eventually put in the work at home and met their goals at the next assessment (Physical Therapist 7)
			Diffusing difficult situations	In the beginning of my career, I worked with an older gentleman who would always say inappropriate things when I was performing manual techniques to his lower back and hip. Following, the second session of brushing it off, I sat down and spoke with him regarding the inappropriate comments. He apologized. I did manual less frequently, but in the future, he didn't make comments and we were able to resolve his pain successfully with therapy. It allowed me to address the issue with other gentlemen more directly (Physical Therapist 3)
			Patients who want personal relationships	I had a patient who wanted to hang out outside of work. I explained that I felt it was inappropriate based off my own previously established work-life boundaries. She accepted my explanation and completed her scheduled visits without a significant change in rapport (Physical Therapist 4)
6	A time when you had to prioritize self-care over the care of others or the care of others over self-care	Self-care	Sacrifice with guilt	We have patients that come from out of state on occasion and in order to keep all clinic visits on the same day, I may have to give up lunch. It can make me irritable but it’s not an everyday occurrence. In terms of vice versa, when I travel, my boss has to see patients. She has to manage our clinic and a program across the entire company, so it tends to evoke a guilt feeling in me (Physical Therapist 2)
	Your reflection on the last 3 months			I committed to attending a dinner after work hosted by my best friend. It was over an hour away but I knew it meant a lot to her, so I said I would go. Unfortunately, that day at work was extremely busy. I worked all day and knew I needed to work for two to three more hours from home. I was drained and could not imagine making the long drive and not getting the rest of my work done. I had to make the hard decision of backing out of dinner, in order to listen to my body/mind and rest at home while finishing work and getting to sleep at my normal time (Physical Therapist 6)
			Actionable self-care	My job has been changing such that the list of things I need to complete keeps getting longer and the number of people I’m responsible for does, as well. This has definitely caused me challenges to choose between caring for the well-being of my staff over myself. I’ve had difficulty during the week carving out time for myself, however, I’ve been more committed to setting boundaries so that I don't do work on the weekends and can enjoy myself and complete more self-care and rest (Physical Therapist 7)
				I was able to care for myself while caring for another by choosing to not let my emotions take over. I envisioned myself as a superhero, stood tall, smiled, and enjoyed the chaos, instead of shutting down and only looking for a solution. A superhero is open to all possibilities, not limited by a lack of resources or the fact that everything is far from the plan (Physical Therapist 8)
			Work-life integration	I had to give up the normal time in the day I take for myself to take care of stuff around the house, as well as my son and husband. By the end of the week, I felt completely exhausted and overwhelmed because there were chores that still needed to be finished, my sleep was compromised, my time was limited so I missed regular exercise, and I had to leave work early so I was behind on documentation. I realized how easy it is to get caught up in life and in helping out those around you, you completely forget to take time to check in with how you are doing. Luckily, I was able to quickly utilize some of the mindfulness strategies to get me through an oddly hectic week. The business of my schedule is not plausible to maintain long term, so if the time frame would have been longer, I would have needed help or had to give somewhere to find time for myself (Physical Therapist 3)
			Effects of stress and anxiety on personal health	I often find myself pushing off things I need to do for myself to keep stress off my wife and kids. I’ve recently been dealing with hypertension related to stress and anxiety. I’ve consulted with my primary care physician and we agreed that I would get back to exercising and eating right and decreasing my focus on all the myriad of things that need to get done on the house. I have made attempts at this with some success, but every time I start to focus on something to decrease my stress or something to relax, I am presented with the knowledge of a stressor in my family’s life. While I need to prioritize my health, I always end of choosing to relieve my family’s stress, which in turn adds to my overall load (Physical Therapist 4)

There also were several important themes and subthemes that emerged upon exploring the 4Ds of appreciative inquiry (Table 5). Reflections on meaning in physical therapy during session one evoked the themes of *patient education, motivation, and adherence*, *clinical operations and patient care*, *personal and professional growth*, and *work-life integration*. *Barriers to providing care*, *documentation*, and *productivity and billing* were some of the underpinnings of the *clinical operations and patient care* theme. *More time for teaching*, *more time to engage and learn from colleagues*, and *more time for research* were some expressions of the physical therapists’ goals for greater *personal and professional growth. Pay and student loan debt* were linked to the theme of *work-life integration*. Physical therapists’ in this study desired greater pay to alleviate debt-related stress and reduce the need for maintaining multiple jobs. Similar to session one, the situational- and self-awareness topic area in session two evoked the themes of *management of patient care* and *physical therapy operations*. *“Conflict between professionalism and morals, and impact on rapport (Discovery) (Physical Therapist 4),”* a desire for *“improved ethics in practice (Destiny) (Physical Therapist 6),”* and *“more consistent internal feelings regardless of the patient interaction to produce more mental stability and clinical presence (Destiny) (Physical Therapist 2)”* reflected the *ethics and morals* and *mental stability and clinical presence* subthemes of *communication with patients*, another theme for session two. *Negative emotions, fostering a supportive environment during patient recovery, more time and resources, the desire for organizational receptiveness and to feel present, stimulated, challenged, part of a team,* and *suggestions for education and team building* were the many components of the physical therapists’ appreciative inquiry reflection on the topic area of compassion fatigue/burnout during session three. Within the topic area of compassion fatigue/burnout, Physical Therapist 5 expressed the *impact of clinician quality of life on patient care*, *“More compassionate, happy healthcare workers, which leads to better care and patient outcomes (Destiny).”* Session four on implicit biases gathered thoughts on *cognitive bias*, *provider’s goals versus patient’s goals*, *patient’s biases*, and *physical therapist’s biases*. Thoughts about establishing boundaries and managing conflict using the 4Ds in session five were categorized *between the front desk, supervisors, and clinical staff*, *between co-workers*, *between physical therapists and patients*, and *between management and physical therapists*. Finally, when reflecting on self-care in session six, *difficulty prioritizing self/limited personal time* was paramount.

**Table 5.** Appreciative inquiry reflective writing

Session	Topic area	Theme	Exemplar quote	
1	Meaning in physical therapy	Patient education, motivation, and adherence	Having to convince patients that physical therapy can help and that they [the patients] have to do a home exercise program (Discovery). Patients could already know something about physical therapy, patients understand they have to put in effort, patients value what physical therapy could do to help them help themselves (Dream). Have patients get information about physical therapy ahead of time, have patients understand their diagnosis (Design) (Physical Therapist 1)	
			When I perceive secondary gains or willful lack of compliance (Discovery). Structure conversations with patients about secondary gains (Design) (Physical Therapist 2)	
			Patient compliance with weight-bearing status/discharge recommendations, refusing to take medical advice (Discovery) (Physical Therapist 5)	
			Parents that follow through and encourage their kids (Discovery). Parents that feel safe, to be honest knowing that I support them, parents arrive on time and have a set time to work on goals with their kids (Dream) (Physical Therapist 8)	
		Clinical operations and patient care	**Subtheme**	**Exemplar quote**
			Barriers to providing care	Having to try to get them [patients] better in fewer visits due to insurance (Discovery) (Physical Therapist 1)
				Insurance limitations, financial limitations (Discovery). No financial barriers for patients (Dream). Treat patients to point of recovery (Design). Less stress about undertreated patients (Destiny) (Physical Therapist 2)
				Insurance denials and hoops to jump through (Discovery). Insurance listened to my recommendations (Dream) (Physical Therapist 5)
				Ability to see patients for however long it is appropriate - not what’s dictated by management/insurance (Dream) (Physical Therapist 6)
			Communication of worth with the broader healthcare team	Convincing patients that I know as much as or more than the medical doctor (Discovery). Front desk and management would respect knowledge and time for physical therapists to perform all aspects of their jobs, less frustration by all, and more understood (Destiny) (Physical Therapist 1)
				Lack of respect from non-therapy staff (Discovery). Not being questioned multiple times by other staff (Dream) (Physical Therapist 5)
				Push-back from other providers (Discovery) (Physical Therapist 6)
			Documentation demands	More concise documentation requirements, getting rid of the expectation of “point of service” documentation (Dream). Create a universal electronic medical record system with only necessary items, all students can be trained using the same system to improve compliance with insurance requirements and quality of documentation (Design). Reduced stress/pressure to do documentation during patient care, leading to better interactions, better outcomes for patient and therapist (Destiny) (Physical Therapist 6)
				Documentation demands over patient care (Discovery). Emphasis on patient care/quality with time to document afterward (not at point of service) (Dream) (Physical Therapist 7)
			Productivity and billing	Having less time with patients due to needing to meet productivity/charge goals (Discovery) (Physical Therapist 1)
			Delegation and limited follow-through	Requirement to delegate the majority of patients to physical therapist assistants, thus limiting my ability to follow patients from evaluation to discharge closely (Discovery). Increase the number of physical therapists to physical therapist assistants to allow for more appropriate delegation (Dream) (Physical Therapist 6)
		Personal and professional growth	**Exemplar quote**	
			Becoming complacent, not having mental challenges weekly/daily to grow (not being involved in the community or research review with colleagues), not having time to be reflective or learn from my mistakes (Discovery) (Physical Therapist 3)	
			Having a career ladder or clear way to advance, having a job with more variety, not just patient care, writing, marketing, meetings, education, and travel (Dream). Express interest in career growth/variety to current boss/employer, find more opportunities outside of work to use other parts of brain, find new job (Design) (Physical Therapist 7)	
			Listen effectively, increase my emotional intelligence to inspire confidence and collaboration (Design) (Physical Therapist 8)	
			**Subtheme**	**Exemplar quote**
			More time for teaching	I could be a Feldenkrais practitioner and teach or present more (Dream). Work part-time to allow for teaching (Design) (Physical Therapist 2)
			More time to engage and learn from colleagues	Learn from or engage with colleagues on a more regular basis (Dream). Having weekly discussions of challenging patients to get input from colleagues, to learn about a specific diagnosis that is challenging you or educate peers, shadow peers with specialties in certain areas to improve patient care (Design) (Physical Therapist 3)
			More time for research	To have more time to research a patient’s diagnosis (Dream). Having one to two hours weekly to contribute to research versus patient care (Design) (Physical Therapist 3)
			Advanced education	Increase skillset, go back to school to have more background/understanding of management (Design) (Physical Therapist 7)
			Community involvement	Be more involved in the community (Dream)(Physical Therapist 3)
				Provide English as a second language class information to parents through work or at work (Design) (Physical Therapist 8)
		Work-life integration	Overextended	Long hours, two jobs, limited days off (Discovery). Force myself to take time off, don't feel bad about saying no (Dream). Take one day off a week and take care of myself before others (Design). Have more energy to give to others, be less irritable, be able to enjoy both work and home life more (Destiny) (Physical Therapist 5)
			Pay and student loan debt	Pay increased, student loan forgiven, or better loan repayment help from job (Dream) (Physical Therapist 7)
			**Exemplar quote**	
			More work-life balance (Destiny) (Physical Therapist 2).	
			Lack of career growth and mobility, feeling like there are other skill sets or things I can do outside of patient care/notes (Discovery) (Physical Therapist 7)	
2	Situational- and self-awareness	Management of patient care	Poor medical management and care for patients referred to physical therapy (e.g., open wounds not being treated, unsafe home environment) (Discovery). Ideally, the patient would be heard and fully examined on the first visit with the primary care physician and referred appropriately versus coming to therapy with multiple long-term issues that are poorly managed (Dream). Patients receiving better, multi-disciplinary care, diagnoses not getting overlooked or untreated secondary to complex cases (Destiny) (Physical Therapist 3)	
			Patients not being up and out of bed ready for therapy when I or others arrive to take them to the gym (Discovery). Registered nurse gets appropriate hand-off from the night registered nurse and makes adjustments to ensure all medications are given in a timely manner, certified nurse assistant starts to get people ready, gets appropriate assist from nursing staff for more dependent patients, certified nurse assistant first helps those with early therapy, appropriate registered nurse/certified nurse assistant staffing to foster success (Design). Medications are given on time allowing patients to better tolerate therapy, decreased missed time, increased patient satisfaction, patients up and ready on time allowing for more targeted/individualized therapy, better outcomes in decreased time, shortened length of stay, and increased revenue (Destiny) (Physical Therapist 9)	
			**Subtheme**	**Exemplar quote**
			Patient expectations/ misconceptions	Working with a patient that doesn't want to work on an issue or do therapy, having a patient believe you can “fix them” and they don't have any responsibility (Discovery). Have discussions with patients at the start, before the session, outlining that they don't have to do physical therapy just because their medical doctor told them to, having an honest conversation that they will get out of therapy what they put into it (Dream). The choices patients have with doing or not doing therapy and have an honest discussion about roles and expectations (Design). Set up the patient for better understanding and accountability, it will take unreasonable expectations off of me (Destiny) (Physical Therapist 1)
				Tension with families and medical doctors, anxiety sensations with tough patients or a tough schedule, feeling like services are not wanted or appreciated (Discovery). Increase education provided to patient/family at start of care about physical therapy, schedule, and participation (Design). Patients/families on board with physical therapy from the beginning and follow the schedule rather than putting off/canceling/complaining, staff are less anxious and patient care improves, thus patients are happier and more likely to participate and appreciate services (Destiny) (Physical Therapist 7)
			Organizational support	Work with good people who are also good therapists (Dream). Try to interview your employer before taking the next position or walk away from those people currently (Design) (Physical Therapist 1)
				Staffing should have the appropriate amount of work to provide care (so much more tension when understaffed) (Design) (Physical Therapist 7)
		Communication with patients	**Exemplar quote**	
			Consistent verbal and non-verbal communication, neutrality, framing results that are accurate without a negative emotional response from the patient (Dream) (Physical Therapist 2)	
			**Subtheme**	**Exemplar quote**
			Ethics and morals	Conflict between professionalism and morals, and impact on rapport (Discovery). Professionally, the conversation during treatment would not extend to deeper issues, such as race relations; morally, we continue into the topic and resolve differences in opinion (Dream). A synthesis of the professional and moral “dreams,” maintain professionalism and limit sensitive discussion while offering limited insight (Design). Allow for improved rapport, decrease outward and inward conflict, limit obstacles to care, address moral dilemmas (Destiny) (Physical Therapist 4)
				Improved ethics in practice (Destiny) (Physical Therapist 6)
			Mental stability and clinical presence	Grounding in the moment, acknowledge my own response to behavior while staying as emotionally neutral in my outward response (Design). More consistent internal feelings regardless of the patient interaction, producing more mental stability and clinical presence (Destiny) (Physical Therapist 2)
		Physical therapy operations	**Exemplar quote**	
			Start every evaluation on time (Design). Set up the patient for better understanding and accountability, it will take stress and burden off of me to try and make up for my co-workers’ shortcomings (Destiny) (Physical Therapist 1)	
			When covering for our outpatient physical therapist, being asked to do progress notes or re-certifications for patients with incomplete assessments, poor plans of care, and no past progression of skilled treatment up to my standards (Discovery). All physical therapists and physical therapist assistants would hold themselves to the highest standards when performing patient care to avoid these ethical dilemmas for other physical therapists and optimize patient care (Dream) (Physical Therapist 6)	
3	Compassion fatigue/burnout	Negative emotions	**Subtheme**	**Exemplar quote**
			Frustration	Frustration about lack of change or progress - specific to the patient, the patient’s family, or other clinicians (Discovery) (Physical Therapist 2)
				Frustration with patients and co-workers (Discovery) (Physical Therapist 5)
			Exhausted	Compassion fatigue sets in for me when I become very invested in a patient. I don't feel I have tools to help a patient or they blame me for an exacerbation of symptoms. At the end of the day, I am exhausted from the exchange (Discovery) (Physical Therapist 3)
				When tired, decreased focus, computer work and care coordination, eye strain, dryness, back pain (Discovery) (Physical Therapist 8)
			Irritable	Physical and mental fatigue, feeling like doing the same thing over and over, feeling like nothing is going to change, being irritable with other staff and residents, feeling like what I’m doing isn't going to make a big difference-the patient will fall again or go back to how they were after therapy is over, other team members become annoying (Discovery) (Physical Therapist 7)
			Helpless	Feeling helpless, feeling decreased compassion for patients, lack of sleep/personal time, increased stress, anxiety, feeling like you are not making a difference (Discovery) (Physical Therapist 9)
			Overextended	I take on too much, calling families, medical doctors… to get the patient what is needed for safety, have multiple days with full schedules, only breaks to pump [for breastfeeding], no time to document (Discovery) (Physical Therapist 3)
				Overextended, need to work two jobs, poorly measured work-life balance (Discovery). Feel like I’m able to work less, balance “professional time” with personal time (family, recreation) (Dream). Decrease hours at second job, allocate more time to “non-work” activities (Design) (Physical Therapist 4)
		Fostering a supportive environment during patient recovery	**Exemplar quote**	
			Patients allowing themselves to recover in their own time, family not pressuring the patient to feel something they don't, other clinicians and I being open to patients having an atypical symptom presentation, and/or the possibility of having an issue “that rarely happens” (Dream). Supportive clinical environment at its best, patients embracing their recovery path with better support from family, support people, and open-minded clinicians (Destiny) (Physical Therapist 2)	
		More time and resources	Wish I had more time for patient care, allotted time to document after patient care, two to three five to ten-minute breaks to decompress and reflect on patients, resources more readily available when more serious situations occur (Dream) (Physical Therapist 3)	
		The desire for organizational receptiveness and to feel present, stimulated, challenged, part of a team	Being present during all patient treatments and providing the best care possible no matter the time constraints (Dream). Adding treatments onto my schedule and making them a priority versus seeing patients when I can squeeze them in between meetings (Design) (Physical Therapist 6)	
			Feeling lively and mentally stimulated, challenged to improve skills, having new opportunities at work, projects, types of patients, learning, organizational change happens after input is given, staff get along and collectively feel like a team who make a difference (Dream) (Physical Therapist 7)	
			Unending focus, clarity, and joy with computer work, care coordination, and treatment ideas, no dry eyes, parents accepting help that is offered, increased laughter with co-workers (Dream) (Physical Therapist 8)	
		Suggestions for education and team building	More burnout education in school, education for staff on what physical therapy is for, more consistent check-ins from managers (Design; Physical Therapist 5)	
			Have regular scheduled learning sessions with team members, team building activities for both work and personal lives (Design) (Physical Therapist 7)	
		Impact of clinician quality of life on patient care	Better quality of life for clinicians, leading to better patient care, lower stress levels (Destiny) (Physical Therapist 3)	
			More compassionate, happy healthcare workers, which leads to better care and patient outcomes (Destiny) (Physical Therapist 5)	
			Engaged, happy employees at work who feel like they matter both to the organization but also to the individual work they do by producing meaningful outcomes, improved morale among team to better weather challenges that will eventually arise (Destiny) (Physical Therapist 7)	
4	Implicit biases	Cognitive bias	I let my perceptual bias and past experience with other patients steer my feelings before ever finding out what was going on with the patient, it appears that the patient was right and no one was listening to her to “help her” (Discovery). Try to go into each evaluation without bias, truly listen to why patients keep leaving other physical therapy clinics (Dream) (Physical Therapist 1)	
			Bias about diseases (uncontrolled diabetes, fibromyalgia, malingering) (Discovery). Withhold attitude and feelings from the session which develop in response to patient behavior, condition, or presentation (Design) (Physical Therapist 7)	
			Not clouded or biased by past treatments/performance/etc (Destiny) (Physical Therapist 7)	
			How my biases affect subjective questions and test selection (Discovery). More open-ended questions, more ways to phrase/communicate to get the most accurate picture of a person’s pre-injury level of function (Design). Optimal care, outcomes match with actual pre-injury level of function, return to play/sport/activity tailored to each person (Destiny) (Physical Therapist 2)	
			Be free of biases going into evaluations and treatments each time, each patient, give patients the opportunity to change, grow, learn each visit without judgment (Design) (Physical Therapist 7)	
		Provider’s goals versus patient’s goals	Having expectations and goals that differ from your patient’s goals or expectations (Discovery). Starting an evaluation/treatment or just interaction with an individual without biases of how you expect your patient to respond to a treatment, what you expect their goals to be, how compliant you expect them to be with exercises, etc (Dream). Hear people out despite their circumstances, do not categorize patients into certain stereotypes, and always ask patients their goals/expectations to ensure you are not making your goals their goals (Design) (Physical Therapist 3)	
			Go into each patient interaction with an open mind, always ask patients what they want out of therapy (Design). Approach each patient individually no matter their medical past/history, always make patients the partners so that you both know the end goal (Destiny) (Physical Therapist 1)	
		Patient’s bias	**Subtheme**	**Exemplar quote**
			Harmful/negative toward others	Patients can hold opinions that are harmful/negative towards others and express them inappropriately making staff/other patients uncomfortable (Discovery). We all could learn to respect one another regardless of our differences and express our opinions respectfully versus harmfully (Dream). Instead of automatically developing a negative bias towards someone that we disagree with, engage in a polite conversation. OR if that is not possible politely inform the patient that therapy is a politics-free zone (Design). People of all ages/backgrounds can get along and respect each other regardless of our differences! (Destiny) (Physical Therapist 6)
		Physical therapist’s bias	Demeanor and personality	Bias about patient demeanor and personality (cranky, mean, rude to the therapist and others) (Discovery) (Physical Therapist 7)
			Attitude toward therapy	Bias about patient attitude to therapy (this won't work, “how many more times?,” complain, does not do what is asked for the home exercise program) (Discovery). Openness to all conditions, less easily frustrated/develop feelings in response to patient’s mood/attitudes (Dream) (Physical Therapist 7)
			Age and health	Bias toward patient age and health (not taking care of oneself, accumulation of preventable issues) – either young or old (Discovery). Staying positive and health-promoting despite conditions, health status, mood, or attitudes (Dream) (Physical Therapist 7)
			Gender	Gender bias, I am not trying to be biased, if I think a person identifies or I am not sure, I ask, some people say we should never make “he/she” references (Discovery). Not making reference to gender unless it relates to the physical therapy outcome, go into each session prepared with an open mind (Dream). I feel like in this society we are expected to make snap judgments, but we know that we should take time to digest information to act kindly and appropriately with no bias (Destiny) (Physical Therapist 8)
			Rationale for physical therapy	[The patient] had me convinced his situation was something it wasn't. I was taken off guard by my assumption that everyone who came for therapy came for therapy as opposed to other reasons, such as legal (Discovery) (Physical Therapist 4)
5	Establishing boundaries and managing conflict	Between the front desk, supervisors, and clinical staff	**Exemplar quote**	
			I will ask my supervisor what the expectations are for the front desk regarding paperwork, time told, etc. If needed, I will ask their supervisor what the best way to communicate when these things aren't done. Hopefully, I will actually talk with the front desk and resolve conflict and not need to involve our supervisors (Destiny) (Physical Therapist 1)	
		Between co-workers	When I am in conflict with a co-worker and, not sure what I did to get on their “wrong side,” I feel very defensive and unsure of how to modify my behavior (Discovery). Finding a way to respond, rather than react to accusatory language or non-verbal behaviors would help to keep my own stress reactions in better control or, be less intense (Dream) (Physical Therapist 2)	
		Between physical therapists and patients	Conflict with patients about compliance, relationship of health prevention and health issues-negative encounters with patient drop off, boundaries with work and professional life (Discovery). Patients should be receptive to instruction and seek treatment due to desire to improve rather than because told to by physician, open to behavior change, open to instruction (Design). Patients will vary in reception but likely respond better when effective communication and expectations are established early in relationships, therapist provide feedback according to patient needs and personal factors which are discussed and considered in care (Destiny) (Physical Therapist 7)	
			**Subtheme**	**Exemplar quote**
			Discriminatory and offensive behavior from patients	When I worked as an aquatic therapist, a gentleman was using racial slurs and complaining about getting in the pool with African American and Mexican women (Discovery). Individuals who don't have prejudices or discriminations against people based off different world views, treating individuals with respect despite gender or race (Dream) (Physical Therapist 3)
				A patient that was rude, verbally aggressive, and sexist (Discovery). Mutual respect between all patients and therapists, regardless of the situation, background, etc (Dream). Allowing patients to share their expectations of the therapist and their background early in the therapeutic alliance and vice versa (Design). Clear communication between patient/therapist that is respectful and healing regardless of the situation/personal differences/background (Destiny) (Physical Therapist 6)
			Patients not ready for discharge	I was discharging a patient who experienced a plateau, no improvement for weeks. I discussed this with her and set a discharge date. On the date, I mentioned the discharge and she did not take it well. I explained her progress and recent plateau offering one to two visits to transition to a home program. She ended up leaving and threatening me with a complaint (Discovery). Longer lead up to discharge, reminder at second to last visit, wait to broach subject until later in session (Design) (Physical Therapist 4)
			Exemplar quote	
		Between management and physical therapists	Boss didn't respect my opinion or boundary, made a joke of it, and moved on. I felt resentful and unsafe in my work environment, work and defending someone else was more important than me (Discovery). Open discussion on clinical boundaries so everyone is aware of each other’s limitations to have a more open, encouraging atmosphere (Design) (Physical Therapist 5)	
			Conflict with management over productivity, hours, care provided, not feeling heard or respected (Discovery). Management less dictatorial and more compromising about staff productivity, more patient-centered, more engaging, and receptive to ideas and input (Dream). Management should provide guidance but also opportunities for involvement and problem-solving to create solutions, rather than black-and-white rules without methods to achieve goals (Design) (Physical Therapist 7)	
			Staff is less anxious, easier going, listen and follow without needing repetitive instruction or reminders, staff more autonomous and receptive (Dream). Staff should be open to feedback and feel comfortable with discussions, should be eager to please employer and succeed and improve processes as well as patient care (Design). Staff will seek out opportunities to provide feedback and ideas for improvement, staff will respect business aspect of the industry and need to meet markers for productivity, employees will have personal factors that influence relationships with management and attention to detail but will be receptive to instruction and feedback when provided calmly and without attacking or making feel defensive (Destiny) (Physical Therapist 7)	
6	Self-care	Difficulty prioritizing self/limited personal time	Balancing personal time with patient need (e.g., travel for weeks at a time) (Discovery). Being able to leave clinic for extended time (travel) without a drop-in patient care and without stressing another clinic staff/clinician (Dream). Find/develop other physical therapists that have similar treatment philosophies and approaches at other clinics or clinicians that can flex in easily and are accepted by the medical providers in the clinic (Design) (Physical Therapist 2)	
			I have difficulty prioritizing myself over people I care about (Discovery). Complete all tasks, let people complain until they do things themselves, stop setting timelines for completion (Dream). I plan to finish everything while not worrying as much (Design). Less stressful, if successful (Destiny) (Physical Therapist 4)	
			I would be able to prioritize myself without letting others down (Dream). Incorporating regular self-care to try and prevent this type of burnout so I can be healthy and maintain my obligations BUT not having to feel guilty if I must prioritize myself (Design). I will reduce my burnout via self-care to be the best person, therapist, and friend I can be (Destiny) (Physical Therapist 6)	
			Prioritizing myself versus work, boundaries each day, job demands/deadlines and new issues infringing on personal time, wanting to please my boss, wanting staff to be happy, wanting work/staff to be organized (Discovery). Consistent boundaries, less stress/less work at home if better identify everyday tasks and time to start/not end of day, self-care more consistent and will feel better when at work to manage stress and unplanned events, regular sense of satisfaction or things to work on to improve workplace and meet demands (Destiny) (Physical Therapist 7)	
			**Subtheme**	**Exemplar quote**
			Managing overwhelming responsibilities	Having too many responsibilities in a short time to be able to take time to ensure your health and spirit are in a good place (Discovery). Despite a busy schedule and other people depending on you to complete tasks or care for them, allot a small amount of time for quick self-care such as deep breathing for three to five minutes or a body scan (Design). I will learn where and when it is okay to give at work or home in order to have time for myself. I have found it very helpful to have some mindfulness strategies to just take a few minutes to reset/organize my thoughts/actions in the midst of everyday chaos to manage these stressful bouts more efficiently (Destiny) (Physical Therapist 3)

4Ds=discovery or “what is;” dream or “what might be;” design or “what should be;” destiny or “what will be [28].”

### Integration

The narrative and appreciative inquiry reflections (Tables 4 and 5) further emphasized the significant differences in the intervention group’s pre- to post-scores for work engagement, mental health, and moral distress (Table 3). As an expression of how the MBT intervention positively impacted overall well-being, Physical Therapist 4 wrote, *“I’ve been working on maintaining awareness of my emotions, where they come from, and what they’re truly reflective of to avoid misplacing them. Also, mindfulness to address my general fatigue, emotional and physical”* in response to the narrative prompt for the session on compassion fatigue/burnout. For the appreciative inquiry reflection on situational- and self-awareness, Physical Therapist 2 committed to *“Work on grounding in the moment, acknowledge my own reaction or response to behavior while staying as emotionally neutral in my outward response (Design).”* Using the 4Ds in the session on self-care, Physical Therapist 3 shared, *“I have found it very helpful to have some mindfulness strategies to just take a few minutes to reset/organize my thoughts/actions in the midst of everyday chaos to manage these stressful bouts more efficiently (Destiny).”*

## Discussion

The goal of this work was to explore the impact of an adapted MBT curriculum [6] in decreasing factors that may contribute to burnout in community physical therapists. We hypothesized that physical therapists would demonstrate higher work engagement, empathy, and job satisfaction, and lower depression, stress, anxiety, and moral distress following the MBT sessions. No differences were detected at baseline across the intervention and control groups except when comparing pre-stress scores. In partial support of this study’s hypothesis, physical therapists in the intervention group demonstrated greater dedication and absorption in their work engagement, improved overall mental health and decreased depression, and reduced moral distress. Absorption demonstrated the greatest change, indicating improved concentration and immersion in occupational duties. The control group did not experience any differences in their pre- to post-questionnaire scores demonstrating that outcomes largely remained unaffected without intervention. Our findings showed that periodic mindfulness strategies can be utilized to promote well-being. In the appreciative inquiry sentiments about “what should be [28],” many physical therapists in this study expressed the intent of incorporating mindfulness strategies into their work. *“…allot a small amount of time for quick self-care, such as deep breathing for three to five minutes or a body scan (Design),”* Physical Therapist 3 wrote when reflecting on self-care. *“…start deep breathing moments with co-workers… (Design),”* Physical Therapist 8 shared in the compassion fatigue/burnout session. In addition to the mindfulness strategies, integration of mindfulness throughout the narrative and appreciative inquiry and small group discussions within each session implied that reflective writing, verbalizing stressors or happy moments related to personal and clinical experiences, and social support likely also contributed to improved well-being.

Sample size, time commitment from participants, and inability to offer continuing education credits and monetary compensation were the differences between this study and the Krasner et al. [6] study. The educational program in mindful communication [6] involved 70 primary care physicians who received $250 to participate in an 8-week intensive phase (2.5 hours per week) inclusive of a 7-hour retreat followed by a 10-month maintenance phase (2.5 hours per month), whereas this work involved 13 physical therapists who received no remuneration to participate in a biweekly training (1.5 hours roughly every 2 weeks) for the duration of 11.5 weeks with no long-term maintenance. This study demonstrated that the basic framework from the Krasner et al. [6] study can be successfully abbreviated and adapted to the everyday lives of participating physical therapists, allowing for minimal disruption and practical application of the mindfulness strategies in between sessions.

Normative or mean values have been established for the TEQ, DASS-21, and MMD-HP. Greater than average empathy [19] at baseline was measured in this study’s combined group of participants, as well as within the respective subgroups, which may help to explain the lack of significant change in pre- to post-empathy scores in the intervention group. Baseline overall mental health scores on the DASS-21 in this study’s intervention group fell within the mid- to high-range of normal. Baseline scores for the intervention group’s mental health subscales fell within the mid- to high-range of normal for depression, the mid-range of normal for anxiety, and the high-range of normal for stress [22,23]. This interpretation helps us to understand why we were possibly unable to impact change on the anxiety subscale, as it was within the mid-range of normal, and why we were able to impact change on the overall mental health score and depression subscale, as they fell within the mid to upper range of normal. However, it does not help further explain why we were unable to impact change on the stress subscale for the intervention group participants. There was a high prevalence of participants with moral distress in this study. Fifty-four percent of the  total sample, 55.56% and 50.00% within the respective intervention and control groups had baseline moral distress scores greater than the average value for healthcare professionals (score = 108.90) [27]. After completion of the MBT, only 14% of intervention group participants still measured greater than the average value for moral distress, and the control group remained unchanged at 50%.

There were a few limitations to this study. The primary limitation was the small sample size. Intervention group participants were recruited across the vast metropolitan area of Phoenix, Arizona. Intervention group sessions were held in person on predetermined dates in the evening during the week, which may have limited the number of physical therapists available to participate. Self-selection bias was introduced by allowing participants to choose whether to take part in either the intervention or control group and likely explains the high stress and moral distress at baseline of the intervention participants. A power analysis was not conducted to determine the sample size in this pilot study; thus, these analyses may be underpowered. How often the participants employed the mindfulness strategies in between study sessions was not prescribed or recorded. Although some qualitative data indicated that participants used the specific strategies while at work, we were unable to report the degree to which this application played a role in the reported outcomes of the study. Investigators were not blinded to the control or intervention group allocation for purposes of scoring the outcomes or conducting the data analysis before the interpretation. Many of the control group participants completed the pre- and post-questionnaires at different timeframes than the intervention group. Accordingly, the variability of the clinical environment during certain times of the year may have impacted the outcomes for the control group. Because of the pilot nature of this study, we did not control for the multiple comparisons, thus introducing a greater likelihood of false positive results. Finally, it is difficult to ascertain if the overall MBT curriculum or a specific component of the curriculum was more impactful on the outcomes that were significant or could have been more impactful on the outcomes that were not significant, as this was not specifically measured or analyzed. Future research should continue to explore the efficacy and effectiveness of MBT and/or other interventions to reduce burnout symptoms and promote well-being with a larger randomized sample of physical therapists. This will help to control for any confounding variables and allow for more robust statistical analysis. Future iterations of similar research should also explore further intervention optimization by distinguishing which aspects of the curriculum were most impactful on the designated outcomes and carry only those components forward in the research progression. Because of the high turnover rate of rehabilitation therapy employees, future work should also include physical therapist assistants, occupational therapists, and occupational therapy assistants [10,11].

The job growth rate for physical therapists and physical therapist assistants is expected to be much faster than average [44,45]. Practicing components involved in MBT to lessen burnout may benefit the well-being of physical therapists while simultaneously enhancing employee retention and improving patient care [6,46,47]. These practices may even be implemented into other facets of physical therapy including the role of a clinical educator where burnout may be prevalent [48]. Human resource departments and benefits committees might consider including MBT as an employee benefit option if it does not already exist, or incentivizing employees who take advantage of MBT as a benefit, if it does already exist. Also, physical therapy education programs might consider integrating MBT throughout their curricula, so these strategies for combating burnout and promoting well-being are acquired early. While these authors recognize that organizational-level factors, like patient load, work hours, and salary, need to be addressed to further positively impact the occupational health of physical therapists, implementing an MBT program demonstrates promise with improving the individual-level factors of work engagement, mental health, and moral distress.
